# Daily melatonin administration improves osseointegration in pinealectomized rats

**DOI:** 10.1590/1678-7757-2017-0470

**Published:** 2018-06-25

**Authors:** Letícia Pitol PALIN, Tarik Ocon Braga POLO, Fábio Roberto de Souza BATISTA, Pedro Henrique Silva GOMES-FERREIRA, Idelmo Rangel GARCIA, Ana Cláudia ROSSI, Alexandre FREIRE, Leonardo Perez FAVERANI, Doris Hissako SUMIDA, Roberta OKAMOTO

**Affiliations:** 1 Univ. Estadual Paulista, Faculdade de Odontologia, Departamento de Ciências Básicas, Araçatuba, São Paulo, Brasil.; 2 Univ. Estadual Paulista, Faculdade de Odontologia, Departamento de Cirurgia e Clínica Integrada, Araçatuba, São Paulo, Brasil.; 3 Universidade Estadual de Campinas, Faculdade de Odontologia de Piracicaba, Área de Anatomia, Piracicaba, São Paulo, Brasil.

**Keywords:** Melatonin, Pineal gland, Dental implants, Tibia, Chronobiological disorders, Sleep disorders, Circadian rhythm

## Abstract

The hypothesis of this study was that the peri-implant bone healing of the group of pinealectomized rats would differ from the control group. The samples were subjected to immunohistochemical, microtomographic (total porosity and connectivity density), and fluorochrome (mineralized surface) analyses.

**Objectives:**

The goal of this study was to investigate the cellular changes and bone remodeling dynamics along the bone/implant interface in pinealectomized rats.

**Material and Methods:**

The total of 18 adult male rats (Rattus norvegicus albinus, Wistar) was divided into three groups (n=6): control (CO), pinealectomized without melatonin (PNX) and pinealectomized with melatonin (PNXm). All animals were submitted to the first surgery (pinealectomy), except the CO group. Thirty days after the pinealectomy without melatonin, the second surgery was conducted, in which all animals received an implant in each tibia (36 titanium implants with surface treatment were installed – Implalife^®^ São Paulo, SP, Brazil). By gavage, the rats of the PNX group received the vehicle solution, and the procedure.

**Results:**

Immunohistochemical analysis for runt-related transcription factor 2 (RUNX2), alkaline phosphatase (ALP), osteopontin (OP) and osteocalcin (OC) showed that the bone repair process in the PNXm group was similar to that of the CO group, whereas the PNX group showed a delay. The microtomographic parameters of total porosity [Po(tot)] and bone surface (BS) showed no statistically significant differences, whereas for the connective density (Conn.Dn) a statistical difference was found between the CO and PNXm groups. Fluorochrome analysis of the active mineralized surface showed statistically significant difference between the CO and PNX and between the CO and PNXm groups.

**Conclusion:**

The absence of the pineal gland impaired the bone repair process during osseointegration, however the daily melatonin replacement was able to restore this response.

## Introduction

Absence of melatonin or its inefficient production affects people who work on night shift, undergo changes in time zone, and suffer from insomnia^16^. In addition, melatonin interferes with bone healing in several ways: through modulation of inflammatory process, collagen fibril formation, osteoblast differentiation, and oxidative stress^6^ ^,^ ^9^ ^,^ ^10^ ^,^ ^15^ ^,^ ^26^ ^,^ ^28^.

A lower production of melatonin, either by genetic modifications or pinealectomy, results in alterations in morphology and bone metabolism^8^ ^,^ ^30^. The literature describes that its action is related to differentiation and increase in osteoblastic activity, as well as osteoprotegerin increase, thus decreasing the action of osteoclasts^18^ ^,^ ^29^. In addition, it can promote bone cell proliferation and increase in production of type I collagen^21^, thus increasing bone mass^19^. In this context, the absence of melatonin has been shown to delay alveolar repair after exodontia^9^ ^,^ ^17^. Melatonin replacement could regulate this alteration, which would emphasize the role of this neurohormone in biological responses that drive bone metabolism, especially in conditions in which a decrease occurs in the circadian release capacity of this hormone by the pineal gland^15^.

With the popularization of dental implants, the understanding of structural characteristics and the reversion of the pathophysiological bone alterations have great importance in increasing the predictability of the success of rehabilitation treatment, considering that characteristics of the bone microarchitecture influence the capacity of the bone in support transmission and the distribution of forces^1^ ^,^ ^9^ ^,^ ^11^.

In addition, although studies have demonstrated the participation of melatonin, when added to biomaterials^3^ ^-^ ^5^, in responses related to bone healing^6^ ^,^ ^26^ ^,^ ^31^, repair of periodontal defects and implants loaded with melatonin^2^ ^,^ ^7^, no studies were performed to evaluate osseointegration during endogenous melatonin deficiency. Thus, the process of how the absence of this hormone interferes with the quality of the perimplantar bone is unclear, which may impair the results of the rehabilitation process in these patients.

Considering this, the aim of this study consists on evaluating the cellular changes and possible complications that may occur along the interface (bone/implant) in pinealectomized rats that received or did not receive daily melatonin replacement as a possible therapy for conditions characterized by a decrease in the secretion of this hormone by the pineal gland.

## Material and methods

### Experimental groups

This study followed the standards of the Ethics Committee on Animal Use (2014/00268) of the Brazilian College of Animal Experimentation – COBEA.

This study was performed in accord with the Animal Research: Reporting of *In Vivo* Experiments (ARRIVE) guidelines^18^, using 18 male rats (*Rattus norvegicus albinus*, Wistar), with three months of age, divided into three groups: CO (control), PNX (pinealectomized without melatonin), and PNXm (pinealectomized with melatonin).

Animals were submitted to the first surgery (pinealectomy) and euthanized 60 days after the second surgical procedure (implant installation in both tibiae). The right tibiae were used for the realization of microtomographic and fluorochromes analyses, and the left tibiae for immunohistochemical analysis.

### Pinealectomy

The rats were anesthetized with ketamine (80 mg/kg b.w., i.m.) and xylazine (10 mg/kg b.w., i.m.). The trichotomy was performed in the scalp region, and the animals were adapted to a stereotactic apparatus. The head skin was disinfected with alcoholic iodine solution; a longitudinal incision and separation of the subcutaneous tissue until the lambda region of the visualization were performed; and scraping of the fibrous joints (serrata) was made among the parietal bones, causing the interparietal bone to be exposed.

After removal of a bone fragment (4.5 mm diameter calvaria, with a trephine drill coupled to a low-speed motor), the venous sinus (region of intersection of the sagittal and transverse sinuses) was visualized. With the aid of tweezers, the pineal gland, which is located just below this sinus, was removed. After the extraction of the gland, the removed bone fragment was placed in its original position, and the animal was detached from the stereotactic apparatus. After hemostasis, the skin was sutured with cotton thread.

As a prophylactic measure after surgery, 0.02 mL of antibiotic (Pentabiótico^®^ Veterinário Pequeno Porte, Fort Dodge Animal Health Ltda., Campinas, SP, Brazil) was injected intramuscularly.

### Implant installation

After 30 days, the 18 rats were submitted to implant installation at the second surgical procedure.

The animals were fasted for eight hours prior to the surgery. They were sedated with the combination of 50 mg/kg of intramuscular ketamine (Vetaset^®^ – Fort Dodge Animal Health Ltda., Campinas, SP, Brazil) and 5 mg/kg of xylazine hydrochloride (Dopaser – Laboratórios Calier do Brazil Ltda., Osasco, SP, Brazil). The rats received mepivacaine hydrochloride (0.3 mL/kg, scandicaine 2% with epinephrine 1:100,000, Septodont, France) as local anesthetic, and hemostasis of the operating field. Trichotomy was performed in the medial region of both tibiae along with the antisepsis using povidone-iodine topical germicide (10% PVP-I, Riodeine Soft Derma Degerming, Rioquímica, São José do Rio Preto, SP, Brazil).

**Figure 1:**
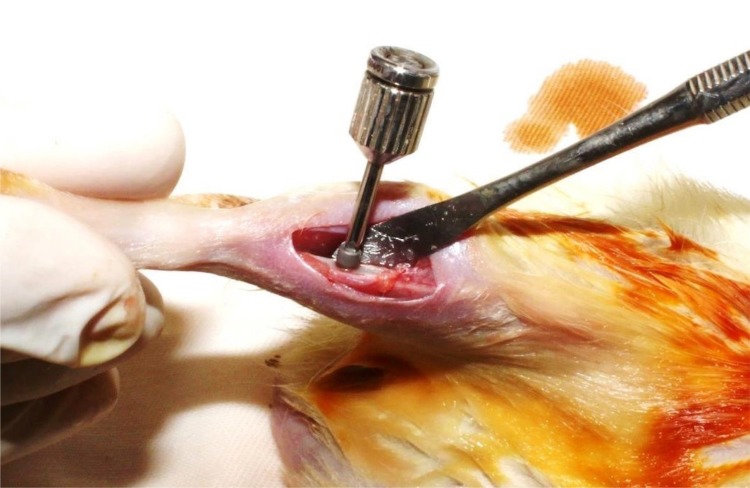
Implant installation in the rat tibia

A 3 cm incision was made with soft tissue avulsion up to the exposure of tibial metaphysis. After that, 36 titanium implants with surface treatment were installed (Implalife^®^ Sistemas Prosthesis Ltda., São Paulo, SP, Brazil). These implants showed in the external hexagonal connection type, with a diameter of 1.6 mm and height of 3.0 mm, using gamma sterilization process. Therefore, the milling was performed with a spiral milling cutter with 1.4 mm in diameter, mounted on an electric motor (BLM 600^®^; Driller, São Paulo, SP, Brazil) at a speed of 1000 rpm under irrigation with 0.9% saline solution (Fisiológico^®^, Biosintética Ltda., Ribeirão Preto, SP, Brazil), and depth of 3.0 mm, with locking and initial stability. The installation was manually conducted with a digital key (Figure 1).

After implant placement, the suture was performed with absorbable suture material (Polyglactin 910 – Vicryl 4.0, Ethicon, Johnson & Johnson, São José dos Campos, SP, Brazil) in the deep plan and with monofilament suture (Nylon 5.0, Ethicon, Johnson & Johnson, São José dos Campos, SP, Brazil) in the external plan. The Pentabiótico^®^ was administered (0.1 mL/kg, Fort Dodge Animal Health Ltda., Campinas, São Paulo, SP, Brazil) in a single intramuscular dose, and metamizole sodium (1 mg/kg/day, Ariston Indústrias Químicas e Farmacêuticas Ltda., São Paulo, SP, Brazil) was administered in the immediate postoperative period.

### Treatment with melatonin

After the surgery for implant placement, animals were treated with exogenous melatonin (Helsinn Advanced Synthesis SA, Via Industria 24, 6710 Biasca, Switzerland) dissolved in saline solution. A volume of 0.2 mL of melatonin at a ratio of 5 mg/kg^13^ was orally administered by gavage to the PNXm group. The PNX group was orally administered a 0.2 mL volume of saline. The administration was performed daily in the late afternoon.

### Application of fluorochromes

At 14 days after the implant installation, 20 mg/kg^24^ of calcein fluorochrome was administered intramuscularly. After 28 days (42 days after implant installation), the red alizarin fluorochrome was administered intramuscularly at the amount of 20 mg/kg^23^.

### Euthanasia

The animals were euthanized at 60 days after implant placement [right tibiae: fluorochrome analysis of the mineralized surface (MS); left tibiae: immunohistochemical analysis] via an anesthesia overdose (sodium thiopental, 150 mg/kg) (Cristália Ltda., Itapira, SP, Brazil).

### Immunohistochemical analysis

For this analysis, the pieces were fixed in formalin, washed in running water, and decalcified in EDTA (10%). Then, dehydration was carried out using a sequence of alcohols. The diaphanization was performed with xylol for later inclusion in paraffin to obtain sections with 5 µm of thickness, and then, they were mounted on slides. The immunohistochemical reactions were used to characterize the osteoblastic phenotype based on the presence of proteins in different stages of osteoblast maturation, starting with the runt-related transcription factor 2 (RUNX2) (pre-osteoblast cells) (SC8788, Santa Cruz Biotechnology, Inc. 10410 Finnell Street, Dallas, TX 75220, USA); alkaline phosphatase (ALP) (SC23430, Santa Cruz Biotechnology, Inc. 10410 Finnell Street, Dallas, TX 75220 USA), showing the beginning of the mineralization process via precipitation of phosphate ions; osteopontin (OP) (SC10593, Santa Cruz Biotechnology, Inc. 10410 Finnell Street, Dallas, TX 75220 USA), which marks mature osteoblasts and the beginning of bone mineralization activity; and osteocalcin (OC) (SC18319, Santa Cruz Biotechnology, Inc. 10410 Finnell Street, Dallas, TX 75220 USA), which is a late protein, considered the marker of bone mineralization, representing the last stage of osteoblast maturation. These proteins were analyzed in a period of 60 days after implant placement.

Immunohistochemical experiments were carried out using immunoperoxidase as a detection method. The Rabbit anti-Goat IgG (H+L) secondary antibody, Biotin (Pierce Biotechnology, Waltham, Massachusetts, USA) was used; the amplifier was the streptavidin (Dako North America, Inc. 6392 Via Real Carpinteria, CA 93013, United States), and the chromogen was the diaminobenzidine (Dako North America, Inc. 6392 Via Real Carpinteria, CA 93013, United States). For each antibody used, the expression of proteins was evaluated semiquantitatively by assigning different “scores”, according with the number of immunostained cells in the wound healing process. The analysis was performed with an optical microscope (Leica DMLB, Heerbrugg, Switzerland) by means of scores (ordinal qualitative analysis), in which the scores had light marking (++), moderate marking (+++), and intense staining (++++). The markings with diaminobenzidine were considered positive, being cautious to hold negative controls for evaluation of the specificity of the antibodies. These scores were established according to Pedrosa, et al.^23^ (2009), Manrique, et al.^19^ (2015), and Ramalho-Ferreira, et al.^24^ (2017): light marking represented about 25% of immunolabeling area in the slices; moderate marking represented about 50% of immunolabeling area in the slices; and intense staining represented about 75% of immunolabeling area in the slices.

### Microtomography (Micro-CT)

For three-dimensional analysis, the left tibiae of groups CO, PNX, and PNXm were removed, dissected to fit the implant installation area, and stored in 70% alcohol. These were first examined via X-ray beam scanning in a computed microtomography digital system. The samples were scanned using a SkyScan^®^ microtomography (SkyScan 1176 Bruker MicroCT, Aartselaar, Belgium) system using 9-mm-thick cuts (50 kV and 500 μ), a copper and aluminum filter, and a 0.3 mm rotation step. The images obtained via projection of X-rays on the samples were stored and reconstituted after the region of interest (ROI) was determined using NRecon software (SkyScan 2011, version 1.6.6.0, Bruker, Aartselaar, Belgium). In the DataViewer software (SkyScan, version 1.4.4 64-bit, Bruker, Aartselaar, Belgium), the images were reconstructed for adjusting the standard positioning for all samples, and observation took place in three planes (transverse, longitudinal, and sagittal). Then, using the CTAnalyser – AWC software (SkyScan Bruker MicroCT version 1.12.4.0, Bruker, Aartselaar, Belgium), an area around the implant (ROI) was defined, bounded by 0.5 mm around the entire implant. This area was defined as the total area (0.5 mm margin around the ROI implants – 4.5 mm × 3.2 mm). The AWC software was then used to analyze and measure the image according to the gray scale (threshold). The threshold had 25-90 shades of gray, which enabled the parameter determination of bone formed around the implants.

The parameters used were total porosity [Po(tot)], connectivity density (Conn.Dn), and bone surface (BS).

### Fluorochrome analysis (active mineralized surface)

After Micro-CT, the right tibiae from the animals in the three groups (CO, PNX, PNXm) were reduced in size and fixed in formalin solution, neutral buffered, 10% (Analytical Reagents^®^, Dinâmica Odonto Hospitalar Ltda., Catanduva, SP, Brazil) for 48 hours and bathed in water for 24 hours. After fixation, the samples were subjected to dehydration with gradually increasing alcohol concentrations from 70%, 90%, and 100%, exchanging the solution every five days with incubation on an orbital shaker (KLine CT-150^®^, Cientec – Laboratory equipment, Piracicaba, SP, Brazil) daily for four hours.

Upon the completion of dehydration, the specimens were immersed in a mixture of 100% alcohol and a Techno Vit^®^ photopolymerizable resin (Heraeus Kulzer, GmbH Division Technique, Philipp-Reis-Str. 8/13 D-61273 Wehrheim, Germany) at different concentrations (100% alcohol + acetone; acetone; acetone + resin; the former being the only resin as immersion medium). The pieces were thus embedded in the Techno Vit^®^ resin, which was photopolymerized and subjected to further processing on the Exakt system.

The cutting and wear of the parts were made in the mesial-distal plane using a cutting system (Exakt^®^ Cutting System, Apparatebau, GmbH, Hamburg, Germany) to obtain about 80-μm-thick sections. Thus, the slices were adapted on the histological blades to continue with other analysis. For quantification of mineralized surface, the blades were scanned with a confocal laser scanning microscope through the longitudinal section from the third until the fifth threads of the implants (Leica CTR 4000 CS SPE, Leica Microsystems, Heidelberg, Germany) (original zoom x100), considered similar to the alveolar bone^14^.

**Figure 2:**
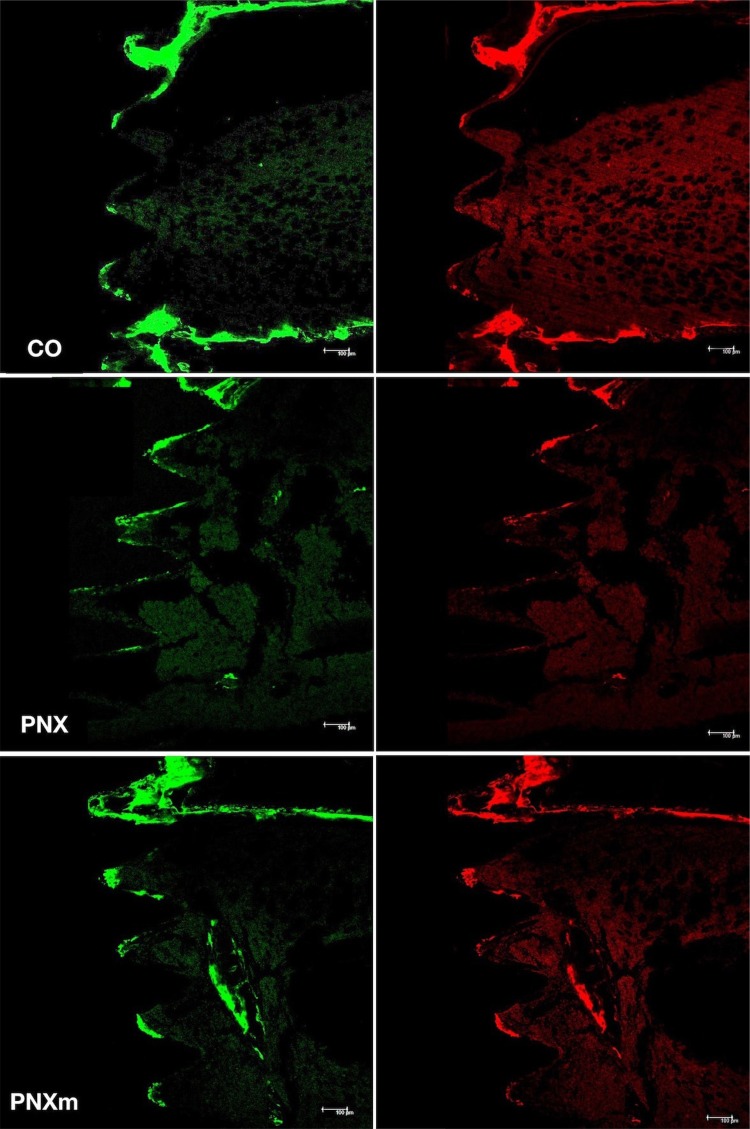
Images of precipitation of calcein (green) and alizarin (red) fluorochromes in Control (CO), pinealectomized without melatonin (PNX) and pinealectomized with melatonin (PNXm) groups

For the quantification of values in the images obtained with the microscope, the ImageJ software (US National Institutes of Health, Bethesda, Maryland, USA) was used. To measure the area of fluorochromes (calcein/alizarin red), the “free hands” tool (μm^2^) was used with a green fluorescent color (calcein) and a fluorescent red (alizarin), thus making it possible to evaluate the mineralized surface in each group (Figure 2).

The extension of the active surface in mineralization at a particular time is given by the total extension of the labeled surface resulting from label administration at that time. Then, the value of the mineralized surface is the value of the alizarin area precipitated divided by the bone surface (result obtained by Micro-CT) of the region of interest^12^.

## Results

### Immunohistochemical analysis

The immunostaining was conducted to characterize the development stage of osteoblasts considering its degree of maturation during the osseointegration process. Thus, the positive markings for RUNX2 (differentiation phase of osteoblasts), ALP (featuring the precipitation of phosphate ions during the mineralization process), osteopontin (extracellular matrix protein related to the initial phase of bone mineralization), and osteocalcin (extracellular matrix protein expressed in later periods, when calcium is precipitated on bone tissue) were evaluated. The purpose of the immunostaining was to evaluate if the drug treatment with melatonin had contributed to the acceleration of the maturity of osteoblasts that actively participate in bone repair responses.

The used chromogen, diaminobenzidine, promoted a brownish coloring in cells that showed positive staining for each protein of choice. It is worth mentioning that osteocalcin, osteopontin and ALP, being extracellular matrix proteins of bone tissue, were also considered positive for the allocation of scores, with presence of proteins in the bone tissue component, some of them are specified below.

The immunohistochemical evaluation was performed through ordinal qualitative analysis, in which immunostaining for different proteins signaling the process of bone formation was characterized based on the allocation of scores.

**Figure 3:**
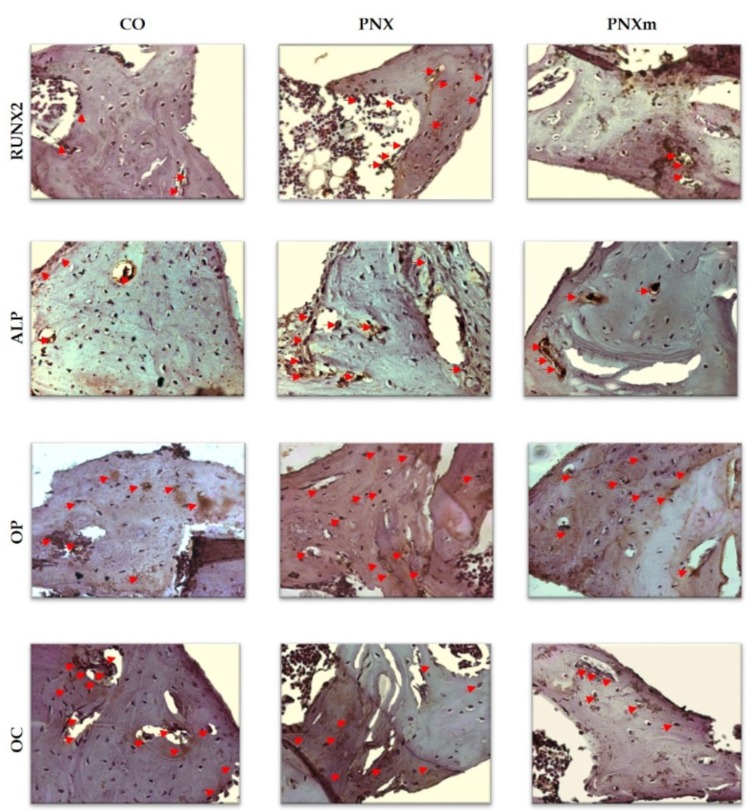
Runt-related transcription factor 2 (RUNX2), alkaline phosphatase (ALP), osteopontin (OP) and osteocalcin (OC) immunostaining at 60 days for the experimental groups. Immunolabeling is indicated by red arrows. (Original, 250x)

**Figure 4:**
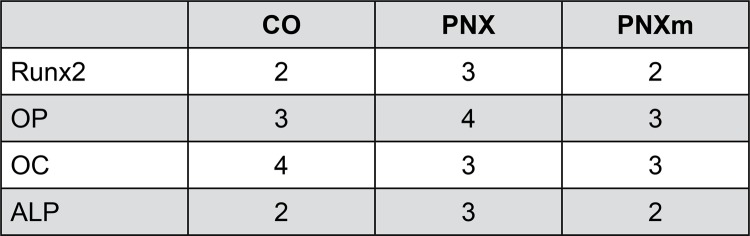
Runt-related transcription factor 2 (RUNX2), alkaline phosphatase (ALP), osteopontin (OP) and osteocalcin (OC) immunostaining at 60 days for the experimental groups

At 60 days, the staining for RUNX2 in the CO and PNXm groups were light (++), indicating a lesser osteoblastic differentiation. The staining for the PNX group was moderate (+++), showing that the bone tissue in this group had more pre-osteoblasts (differentiation activity in progress, which may represent an early stage in the process of bone formation) (Figures 3 and 4).

At 60 days, the staining for ALP in the CO and PNXm groups were light (++) in the bone matrix. However, the PNX group had moderate staining (+++) due to the greater presence of connective tissue (Figures 3 and 4).

The osteopontin expression in the CO and PNXm groups was moderate (+++) around the spirals of the implants, and intense (++++) in the PNX group, as the large amount of bone matrix did not mineralize, characterizing a delay in the bone tissue mineralization process (Figures 3 and 4).

Staining for osteocalcin in the CO group was intense (++++), characterizing the process of mineralization and showing an advanced degree of bone maturity. In the PNX and PNXm groups, the staining of OC was moderate (+++) in the bone matrix, showing a lower degree of the maturity of the bone formed around the spirals as compared with the CO group (Figures 3 and 4).

### Microtomographic analysis

**Figure 5:**
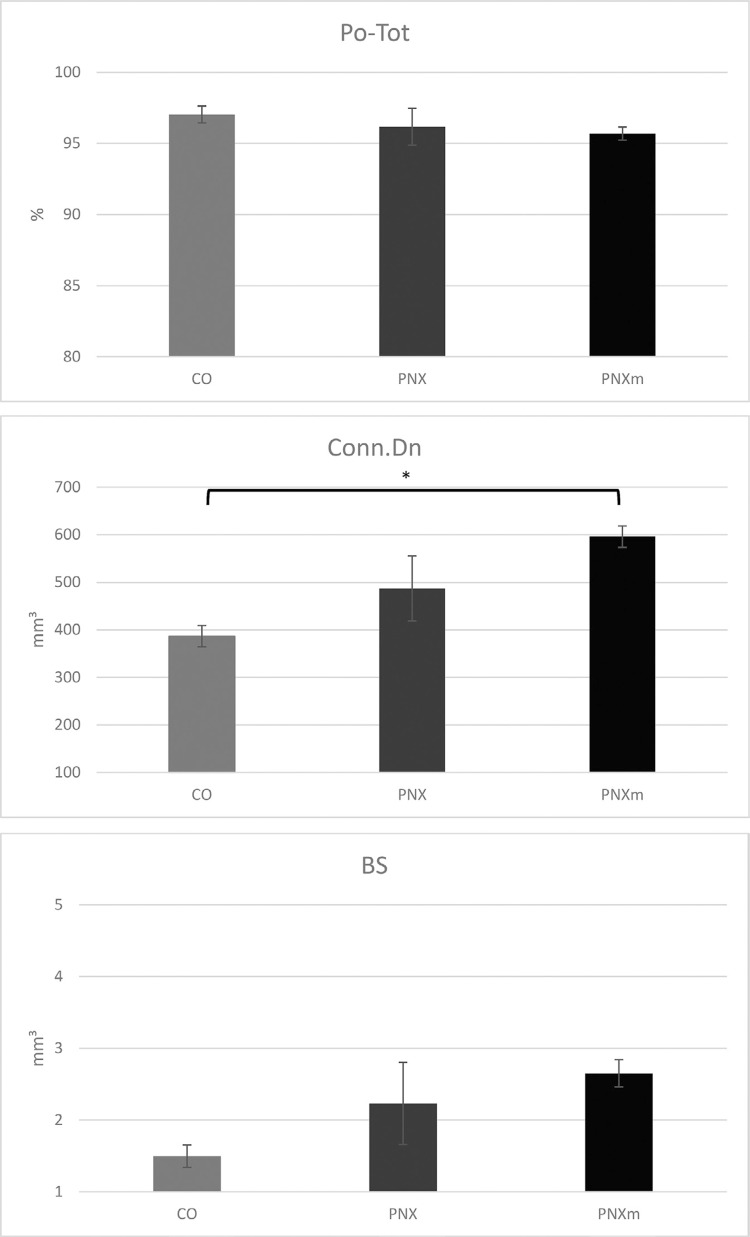
Graphic showing total porosity [Po(Tot)], connectivity density (Conn.Dn) and bone surface (BS) results. [Po(Tot) and BS parameters had no statistically significant difference between the groups (p>0.05); Conn.Dn parameter: CO (386.80)<PNXm (596.20), p=0.0332]. *p<0.05

Considering the parameters of Po(tot), Conn.Dn, and BS, the results were subjected to the Shapiro-Wilk test (p=0.5652) and then were submitted to the parametric analysis of variance (ANOVA) and Tukey’s *post hoc* test. Statistical results showed that a statistically significant difference was found only for the Conn.Dn parameter between the CO and PNXm groups, in which CO (386.80)<PNXm (596.20), p=0.0332 (Figure 5).

For the parameters of Po(tot) and BS, no statistically significant difference was found between the groups (p>0.05) (Figure 5).

### Fluorochrome analysis (active mineralized surface)

**Figure 6:**
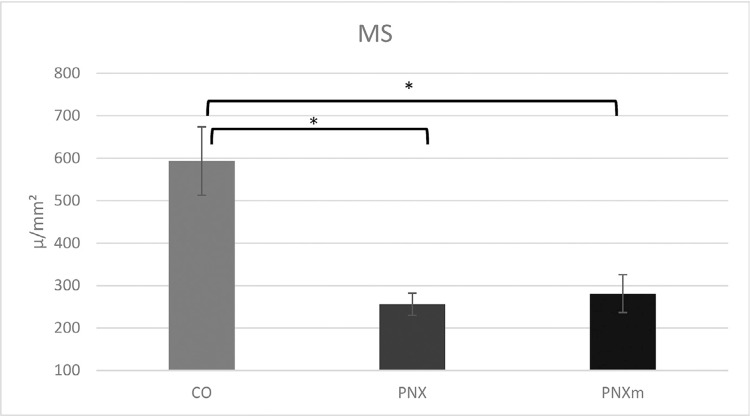
Graphic showing mineralized surface (MS) results [CO × PNX groups in which CO (391,90)>PNX (232.04), p=0.0058; CO × PNXm groups in which CO (391.90)>PNXm (267.05), p=0.0168]

Results of the mineralized surface were submitted to the Shapiro-Wilk test, and then, the parametric ANOVA test was used with Tukey’s *post hoc* test. The comparison between the groups showed that a statistically significant difference was found between the CO and PNX groups [in which CO (391.90)>PNX (232.04), p=0.0058] and between the CO and PNXm groups [where CO (391.90)>PNXm (267.05), p=0.0168] (Figure 6).

## Discussion

This study evidenced that melatonin treatment is related to the discrete improvement of several parameters of bone quality related to osseointegration success, especially in the condition when the pineal gland has been removed. It is important to highlight that during long periods of melatonin administration, these differences can be better stressed, due to the osteoblast activity that may be driven by extracellular matrix proteins that had better classifications considering melatonin replacement in animals^15^.

Some studies state that melatonin has been linked to osteoblastic differentiation^20^. However, the role of melatonin replacement has not yet been widely evaluated, and no responses regarding osteogenesis around titanium implants have been generated so far^10^. Links between melatonin and bone metabolism have been documented in several studies^4^ ^,^ ^6^ ^,^ ^7^ ^,^ ^26^ ^,^ ^27^ ^,^ ^31^. In these investigations, melatonin acted on the bone as a local growth factor. It is known that bone marrow has high concentrations of melatonin. In addition, Witt, et al.^32^ (2006) showed that melatonin influences bone cell precursors in the bone marrow of rats.

Koyama, et al.^19^ (2002) showed for the first time that the administration of pharmacological doses of melatonin during the growth of young rats increased the cancellous bone mass and pointed to effects mediated primarily through the inhibition of bone resorption. They also concluded that the administration of doses (5 mg/kg or 50 mg/kg) of melatonin appeared to have no significant toxic secondary effects. These findings were relevant; however, these observations need to be confirmed in adult animals, which have no endogenous melatonin, and may help to address the concept of osseointegration, which is one of the reasons for this study. These results^19^ undoubtedly have biological importance and served to support the findings of this research.

In our study, we could observe that in relation to the cell characterization of the osteoblast lineage (via immunostaining for RUNX2, osteopontin, osteocalcin, and ALP), the CO and PNXm groups exhibited similar light immunostaining for RUNX2 and ALP, and moderate immunostaining of osteopontin and osteocalcin. Regarding the PNX group, we found positive immunostaining for the same proteins, however, with a pattern or behavior indicating a delay in the bone healing process. These data are justified by the important presence of the proteins of RUNX2, osteopontin, and ALP in the PNX group. These particular proteins mark the initial events of the bone healing process, at 60 days.

The morphometric analysis performed in this study showed that the Po(tot) was similar among the three experimental groups. On the other hand, the Conn.Dn was higher in the pinealectomy, especially when melatonin was replaced. The quality of the bone formed close to the implants had similar characteristics in relation to porosity and the bone surface. However, the Cnn.Dn showed higher results for PNXm followed by PNX, whereas the CO group had the lowest result in this parameter, which was statistically significant in comparison with PNXm (p<0.05, Tukey’s *post hoc* test), showing that the increase in connectivity of cancellous bone could be an attempt of the body to compensate the lack of minerals caused by pinealectomy, being enhanced with the hormonal repositioning of melatonin. Even though the CO group had the worst result for Cnn.Dn, the MS could prove that it had the highest mineralization among the groups, followed by the PNXm group. We emphasize that the extracellular matrix of the bone showed an increase that could be justified by the increase in the number of immunolabeling proteins of the bone matrix observed in the immunohistochemical results for the CO and PNXm groups.

## Conclusion

Based on the results of this study, we can notice an interference in cellular responses and protein activity in the absence of melatonin, showing a delay in the production of proteins that stimulate bone formation, and the replacement of this same hormone seems to partially restore cellular and protein responses as well as the mineralization process, as observed in the results of fluorochromes. The melatonin replacement, in most parameters evaluated in this study, showed a behavior very close to that of the CO group.

Therefore, the absence of the pineal gland impairs the bone repair process during osseointegration, however the daily melatonin replacement was able to partially restore this response.
